# Genotype–phenotype correlations and functional characterization of novel *PAX2* variants in a 10-patient pediatric cohort

**DOI:** 10.1080/0886022X.2025.2552911

**Published:** 2025-09-15

**Authors:** Meng Fu, Hongjie Zhuang, Yanling Wen, Lizhi Chen, Mengjie Jiang, Yuanyuan Xu, Yanlai Tang, Liping Rong, Xiaoyun Jiang

**Affiliations:** a Department of Pediatric Rheumatology and Nephrology, Sun Yat-sen University First Affiliated Hospital, Guangzhou, China; b Department of Pediatrics, Sun Yat-sen University First Affiliated Hospital, Guangzhou, China

**Keywords:** Children, kidney dysplasia, novel variant, *PAX2*, proliferation

## Abstract

**Background:**

*PAX2* is a key developmental gene, and its mutations are primarily associated with kidney and ocular anomalies, predominantly affecting children. This study aims to analyze the clinical manifestations and genetic characteristics of children with *PAX2* mutations and to assess the functional impact of novel variants.

**Methods:**

Clinical data were retrospectively reviewed in 10 children diagnosed with *PAX2* mutations through whole-exome sequencing from a pediatric hereditary disease cohort database. AlphaFold 3 (AF3) was used for protein structural modeling. Novel variants were functionally assessed via HK-2 cell proliferation assays.

**Results:**

The median age at initial presentation was 4.2 years (IQR 0–6.5). All 10 patients presented with proteinuria or microscopic hematuria. Nine had kidney dysplasia, and five progressed to stage 5 chronic kidney disease. Five patients had *PAX2*-related ocular abnormalities. Hepatic dysfunction and spermatic cord hydrocele were reported as potential novel phenotypes. A total of nine distinct *PAX2* mutations were identified, including five novel variants. AF3 modeling revealed significant conformational disruptions in the novel variants, with root mean square deviation (RMSD) values ranging from 1.1 to 43.6 Å. Functional assays demonstrated that four novel variants significantly impaired the proliferative capacity of HK-2 cells.

**Conclusions:**

This study characterizes five novel *PAX2* variants with confirmed structural (RMSD 1.1–43.6 Å) and functional (HK-2 proliferation impairment) impacts, expanding both the mutational and phenotypic spectra in Chinese children. The findings highlight the association between *PAX2* mutations and early-onset kidney disease with potential extrarenal involvement. Early genetic diagnosis and timely kidney-protective interventions are essential to improve outcomes.

## Introduction

*PAX2* gene (OMIM#167409) is a transcription factor that plays a crucial role in the development of organs such as the kidneys and eyes. Located on chromosome 10q24.3-10q25, the gene consists of 12 exons [1–3]. Exons 2–4 of *PAX2* encode the paired-box domain, exons 5 and 7 encode the octapeptide motif and homeodomain, respectively, while exons 8 and 9 encode the transactivation domain. These domains are critical for the transcription factor’s regulatory activity, DNA recognition, and sequence-specific DNA binding [3]. During early embryonic development, *PAX2* is highly expressed in the intermediate mesoderm at the tail region, where it plays an essential role in kidney development [4]. Studies have shown that *PAX2* homozygous mutant mice exhibit a complete lack of kidneys and are lethal [5,6]. In contrast, *PAX2* heterozygous mutations result in reduced ureteric bud branching and insufficient nephron number, ultimately leading to abnormal kidney development and functional deficits [7]. *PAX2*’s critical role in development is widely attributed to its potent anti-apoptotic activity and its ability to promote cellular proliferation [8,9].

*PAX2*-related diseases are autosomal dominant disorders, with the kidneys and eyes being the primary affected organs [10]. Renal and ocular phenotypes commonly include kidney dysplasia, renal cysts, vesicoureteral reflux (VUR), and retinal abnormalities [11]. In addition to its role in the normal development of the kidneys and eyes, *PAX2* is also expressed in other organs such as the midbrain, cerebellum, spinal cord, otic vesicle, and pancreas. It may also contribute to the development of the skeleton, ovaries, and cardiac morphology [12,13].

*PAX2* mutation-related diseases typically manifest in childhood and are associated with a poor prognosis. These disorders demonstrate significant genotypic and phenotypic heterogeneity, and a clear correlation between genotype and phenotype has yet to be established. Due to the rarity of *PAX2* mutation-related diseases, reports on *PAX2* variants in Chinese children remain limited. Furthermore, the high degree of heterogeneity between the genotype and phenotype of *PAX2* mutations poses considerable challenges for clinicians in recognizing, diagnosing, and managing these conditions.

To address these challenges, we aimed to investigate the clinical and genetic features of *PAX2* mutations in Chinese pediatric patients from our center. In this study, we analyzed the clinical data of children with *PAX2* gene mutations from our center and identified several novel variants along with their associated phenotypes. These findings expand the phenotypic spectrum of *PAX2*-related disorders and underscore the gene’s pivotal role in pediatric kidney disease. By integrating AlphaFold 3 (AF3) structural modeling with *in vitro* functional assays in HK-2 cells, we show that these variants impair cell proliferation, offering mechanistic insights into their pathogenic effects.

## Materials and methods

### Study population

We retrospectively analyzed 10 patients in a pediatric cohort with *PAX2* mutations (NM_003990.4) who were admitted to the Pediatric Rheumatology and Nephrology Center at the First Affiliated Hospital of Sun Yat-sen University between January 2015 and October 2024. Relevant clinical data were extracted from the hospital’s hereditary disease database. All patients were diagnosed with *PAX2* mutations through whole-exome sequencing (WES). The familial inheritance patterns of the mutations were verified by peripheral blood DNA extraction from family members (parents) and subsequent Sanger sequencing for nine patients. This study was approved by the Ethics Committee of the First Affiliated Hospital of Sun Yat-sen University.

### Clinical assessment

The clinical data include demographic information (ethnicity and gender), initial symptoms and age of onset, renal and extra-renal manifestations, genetic testing results, renal pathology features, age at onset of end-stage kidney disease (ESKD), and creatinine clearance rate. Chronic kidney disease (CKD) was classified into five stages based on the creatinine clearance rate. The methods used to assess microscopic hematuria, proteinuria, nephrotic-range proteinuria, and CKD staging are detailed in Supplementary Table 1 [14]. Additionally, we determined whether the variants identified in the patients were novel variants by searching the Human Gene Variant Database (HGMD), the Leiden Open Variation Database (LOVD), and ClinVar and evaluated their pathogenicity according to the ACMG guidelines [15].

### Prediction and structural analysis of mutant *PAX2* proteins

The coding sequence (CDS) and corresponding amino acid sequence of the human *PAX2* gene were retrieved from the NCBI Consensus Coding Sequence (CCDS) database (https://www.ncbi.nlm.nih.gov/projects/CCDS/CcdsBrowse.cgi). For each novel variant, mutant amino acid sequences were generated based on the nucleotide alterations relative to the reference sequence. Both wild-type and mutant *PAX2* protein sequences were submitted to AF3 for structural prediction. The model with the highest predicted confidence score (model 0) was selected for further analysis. Structural visualization and comparative analysis of the wild-type and mutant proteins were performed using PyMOL (version 3.1). Structural perturbations were quantified by calculating the Cα root mean square deviation (RMSD) after optimal superposition. Conformations were considered similar at an RMSD <2.0 Å [16], with higher values indicating greater divergence. While a global RMSD <2.0 Å preserves overall structure, a local RMSD >1.5 Å in functional domains (e.g., DNA-binding regions) may disrupt function [17].

### Cell culture and treatments

The immortalized human proximal tubule epithelial cell line HK-2 (CRL-2190, ATCC, Manassas, VA) was cultured in Dulbecco’s modified Eagle medium/Nutrient Mixture F-12 (DMEM/F-12, 11320033, Gibco, Waltham, MA) and supplemented with 10% FBS (Gibco, Waltham, MA). Cells were maintained at 37 °C in a humidified atmosphere containing 5% CO_2_ and grown to approximately 60–80% confluence. The cells were then transduced with different lentiviral vectors, including those carrying mutational *PAX2* variants, wild-type *PAX2*, and GFP (Yunzhou Biotechnology, Guangzhou, China). After transduction, cells were selected with 2 μg/mL puromycin (P8230, Solarbio, Beijing, China).

### Quantitative real-time polymerase chain reaction (RT-qPCR)

Total RNA was extracted from cultured HK-2 cells using the RNA-Quick Purification Kit (RN001, ESscience, Shanghai, China) and cDNA was synthesized using the Transcriptor First Strand cDNA Synthesis Kit (4897030001, Roche, Basel, Switzerland). RT-qPCR was performed using the FastStart Universal SYBR Green Master (Rox) (4913914001, Roche, Basel, Switzerland), and data analysis was conducted using the 2–ΔΔCT method. Primer information for PCNA, MCM2, and MKI67 are listed in Table 1.

**Table 1.** Real time RT-PCR primer sequences.

Gene (human)	Forward primer	Reverse primer
PCNA	CCTGCTGGGATATTAGCTCCA	CAGCGGTAGGTGTCGAAGC
MCM2	ATGGCGGAATCATCGGAATCC	GGTGAGGGCATCAGTACGC
MKI67	ACGCCTGGTTACTATCAAAAGG	CAGACCCATTTACTTGTGTTGGA
ACTB	CATGTACGTTGCTATCCAGGC	CTCCTTAATGTCACGCACGAT

### Western blotting

Cultured cells were lysed in RIPA buffer. Protein samples were separated by SDS-PAGE and transferred onto polyvinylidene difluoride membranes. The membranes were incubated with the primary antibody at 4 °C overnight, followed by incubation with a horseradish peroxidase (HRP)-conjugated secondary antibody (anti-rabbit IgG, #7074, Cell Signaling Technology, Danvers, MA). Detection was performed using the Immobilon Western HRP Substrate Detection Kit (WBKLS0500, Millipore, Burlington, MA). The primary antibodies used included anti-Flag (ab205606, Abcam, Cambridge, UK), anti-PCNA (10205-2-AP, Proteintech, Wuhan, China), and anti-α-tubulin (HRP-conjugated, #12351, Cell Signaling Technology, Danvers, MA).

### Cell proliferation assay

HK-2 cells were seeded at a density of 5 × 10^4^ cells per well in 6-well plates, with three replicates per group. After attachment, cells were digested and counted to determine the initial cell number at 0 h. Subsequently, cells were digested and counted at 24, 48, and 72 h to construct the cell proliferation curve.

### Statistical analysis

Statistical analysis was performed using GraphPad Prism 9.0 (La Jolla, CA). The Shapiro–Wilk test was used to assess normality, and Levene’s test to evaluate homogeneity of variance. Data with normal distribution are presented as mean ± standard deviation (SD), while non-normally distributed data are expressed as median with interquartile range. Group comparisons were conducted using one-way ANOVA followed by Tukey’s *post hoc* test. A *p-* value <.05 was considered statistically significant.

## Results

### General information

This study included a total of 10 patients, including six males and four females, all of whom were sporadic cases. The median age of symptom onset was 4.2 years (IQR 0–6.5) years (range: from the embryonic period to 11.9 years). Among them, two patients (patients 1 and 8) were referred for evaluation due to kidney abnormalities detected by ultrasound during maternal antenatal screening. Patient 1 presented with right renal hydronephrosis, and patient 8 with left renal atrophy. Two patients (patients 5 and 10) presented with kidney dysfunction, while four patients (patients 3, 4, 6, and 7) were diagnosed based on abnormal urine tests. Patient 2 sought consultation due to increased nocturia, and patient 9 presented with nocturnal enuresis (Table 2). A review of the prenatal histories revealed documented renal abnormalities only in patients 1 and 8, while no relevant prenatal data were available for the other patients.

**Table 2.** Summary of clinical characteristics in 10 pediatric patients with *PAX2* mutations.

	Patient no.									
	1	2	3	4	5	6	7	8	9	10
Gender	M	F	M	M	M	M	F	M	F	M
Onset age (years)	0	5.5	3.6	6.8	Postnatal day	6.4	11.9	0	4.7	1.0
Chief complaint	Kidney ultrasound abnormality	Nocturia	Foamy urine	Foamy urine	Abnormal serum creatinine levels	Edema foamy urine	Edema foamy urine	Kidney ultrasound abnormality	Nocturnal enuresis	Abnormal serum creatinine levels
Onset of ESKD (years)	**–**	7.5	**–**	11.1	3.0	10.9	**–**	**–**	**–**	11.0
MHU	+	+	**–**	+	+	+	+	+	+	+
PU	**–**	NPU	+	NPU	**–**	NPU	+	**–**	+	+
24 h urinary protein (g/24 h)	NA	0.949	0.804	1.206	0.01	1.64	0.412	NA	0.6	0.405
eGFR (mL/min/1.73 m^2^)	82.76	8.69	17.96	5.78	7.72	13.22	111.09	63.7	29.62	4.41
Age at renal ultrasound (years)	0.9	7.3	8.2	6.8	3.7	10.8	12.0	2.2	9.4	12.0
Bilateral renal hypoplasia	+	+	+	+	+	+	–	–	+	+
Unilateral renal hypoplasia	–	–	–	–	–	–	–	+ (left)	–	–
Multiple renal cysts	–	+ (bilateral)	–	–	–	+ (bilateral)	–	–	–	–
Single renal cysts	–	–	+ (right)	–	–	–	–	–	+ (left)	–
Age at renal biopsy (years)	NA	NA	NA	6.8	NA	6.4	12.0	NA	NA	NA
Pathology	NA	NA	NA	FSGS	NA	FSGS	Focal segmental mesangial and endothelial cell proliferation in the glomerulus	NA	NA	NA
*Ophthalmological findings*										
Visual field defect	NA	NA	–	NA	**–**	+ (right)	NA	–	**–**	–
Amblyopia	NA	NA	–	NA	**–**	+	NA	–	**–**	–
Other ocular findings	–	Enlarged physiological blind spot	Enlarged physiological blind spot	–	Indistinct optic disk borders; enlarged optic cup	**–**	–	–	**–**	Optic disk swelling
Other findings	**–**	Growth retardation	Growth retardation	Growth retardation	Growth retardation; right spermatic cord hydrocele	**–**	**–**	Liver dysfunction (hepatic fibrosis)	–	Growth retardation; left-sided hearing loss

F: female; M: male; PU: proteinuria; NPU: nephrotic-range proteinuria; MHU: microscopic hematuria; eGFR: estimated glomerular filtration rate; FSGS: focal segmental glomerulosclerosis; NA: not available.

### Kidney manifestations

Nine patients had renal dysplasia, including eight with bilateral kidney hypoplasia and one with unilateral (left) kidney hypoplasia. Renal cysts were detected in four patients, two of whom were multiple cysts and two with a single cyst. All patients exhibited proteinuria and/or microscopic hematuria. Seven patients had proteinuria, three of whom were classified as nephrotic-range proteinuria (≥50 mg/kg/day). Microscopic hematuria was observed in nine patients (Table 2).

Patients 1 and 5 were evaluated for VUR. Patient 1 was found to have right-sided grade III VUR, with no ureteral dilation observed. Patient 5 had bilateral VUR (left side grade V, right side grade III) with complete dilation of the left ureter.

Five patients (patients 2, 4, 5, 6, and 10) progressed to ESKD, with the onset occurring between the ages of 3 and 11.1 years. Patients 2, 4, 6, and 10 all underwent allogeneic kidney transplantation, with an average transplantation age of 10.4 ± 1.0 years. Three patients underwent kidney biopsy: focal segmental glomerulosclerosis (FSGS) was observed in patients 4 and 6, while patient 7 exhibited segmental mild mesangial and endothelial cell proliferation (Table 2).

### Extrarenal manifestations

Ophthalmological records, including fundoscopic examination and optical coherence tomography (OCT), were relatively complete in six patients. Among them, patient 6 exhibited visual field defects and amblyopia; patient 2 and patient 3 both presented with enlarged physiological blind spots; patient 5 showed indistinct optic disk margins and an enlarged optic cup; and patient 10 demonstrated optic disc swelling. Several patients also presented with relatively uncommon extrarenal manifestations. Patients 2, 4, 5, and 10 experienced growth retardation from infancy. Ultrasonography in patient 5 revealed a right-sided hydrocele. Patient 8 demonstrated hepatic dysfunction, characterized by significantly elevated alanine aminotransferase (ALT: 115 U/L) and aspartate aminotransferase (AST: 165 U/L) levels. Liver biopsy further confirmed the presence of mild hepatic fibrosis. We excluded common etiologies such as viral hepatitis, autoimmune hepatitis, and copper metabolism disorders. Additionally, patient 10 presented with left-sided hearing impairment (Table 2).

### *PAX2* mutations

**Figure 1.**
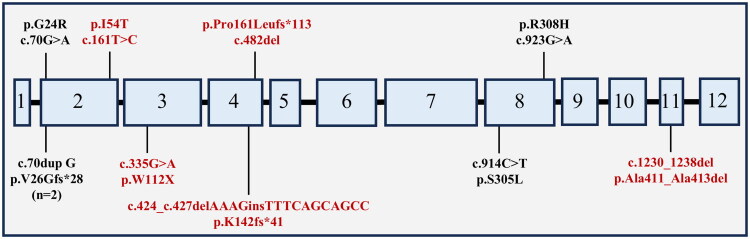
Distribution of *PAX2* mutations in 10 patients. Schematic representation of the *PAX2* gene structure. Boxes denote exons; text outside boxes indicates mutation sites. Novel variants (dark red) are not recorded in the Human Gene Mutation Database (HGMD), Leiden Open Variation Database (LOVD), or ClinVar.

Among the 10 patients, nine distinct *PAX2* gene mutations were identified, with the majority occurring between exons 2 and 4 (Figure 1). The variants c.161T > C, c.335G > A, c.1230_1238del, c.482del, and c.424_c.427delAAAGinsTTTCAGCAGCC were novel and have not been previously reported in HGMD, LOVD, or ClinVar. According to the ACMG guidelines, the c.424_c.427delAAAGinsTTTCAGCAGCC mutation was classified as pathogenic, while c.161T > C, c.335G > A, and c.482del were considered likely pathogenic. The pathogenicity of c.1230_1238del remains uncertain (Table 3).

**Table 3.** Genetic features and variant information of 10 children with *PAX2* variants.

Patient no.	Testing age (years)	Nucleotide change	Predicted effect on protein	Location	Zygosity (segregation)	ACMG standards
1	0.8	c.923G > A	p.R308H	EX8	Het(P)	PS2 + PM2 + PP3 (likely pathogenic)
2	7.3	c.161T > C	p.I54T	EX2	Het(N)	PS2 + PM2 + PP3 (likely pathogenic)
3	8.2	c.70dup G	p.V26Gf s*28	EX2	Het(N)	PVS1 + PS2 + PS4 + PM2 (pathogenic)
4	6.8	c.70dup G	p.V26Gf s*28	EX2	Het(N)	PVS1 + PS2 + PS4 + PM2 (pathogenic)
5	3.7	c.335G > A	p.W112X	EX3	NA	PVS1 + PM2 (likely pathogenic)
6	10.8	c.70G > A	p.G24R	EX2	Het(N)	PS2 + PM1 + PM2 + PM5 + PP3 (pathogenic)
7	12.0	c.914C > T	p.S305L	EX8	Het(M)	BP4 (uncertain)
8	2.2	c.1230_1238del	p.Ala411_Al a413del	EX11	Het(M)	PM2 + PM4 (uncertain)
9	9.4	c.482del	p.Pro161Leufs*113	EX4	Het(N)	PVS1 + PM2 (likely pathogenic)
10	11.5	c.424_c.427delAAAGi nsTTTCAGCAGCC	p.K142fs*41	EX4	Het(N)	PVS1 + PS2 + PM1 + PM2 (pathogenic)

All mutations were heterozygous. Sanger sequencing revealed that six variants were undetectable in first-degree relatives (parents), suggesting these mutations may be *de novo*. The mutation in patient 1 was inherited from the father, while the mutations in patients 7 and 8 were inherited from their respective mothers. However, the parents of three patients exhibited no symptoms, and urine tests showed no abnormalities (Table 3). The parents of patient 5 were not tested, so the origin of the mutation remains unknown.

### Three-dimensional structural features of novel *PAX2* mutant proteins

**Figure 2.**
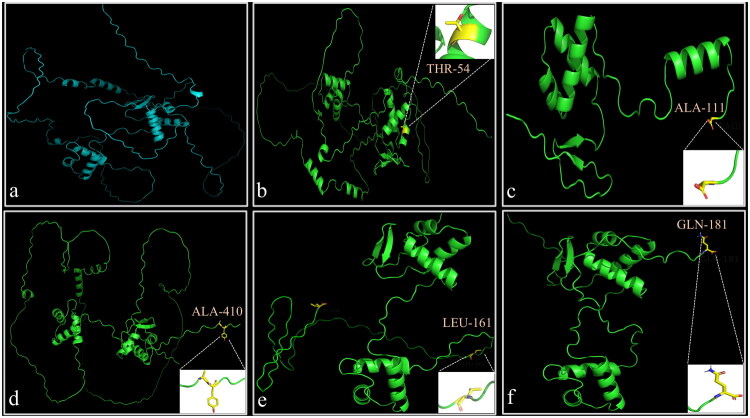
AlphaFold 3 prediction of the three-dimensional structures of wild-type and mutant *PAX2* proteins. (a) Predicted 3D structure of the wild-type *PAX2* protein. (b–f) Predicted 3D structures of novel *PAX2* variants: c.161T > C, c.335G > A, c.1230_1238del, c.482del, and c.424_c.427delAAAGinsTTTCAGCAGCC, respectively, with the corresponding mutated amino acid residues highlighted.

The predicted three-dimensional structures of wild-type *PAX2* and the five novel variants, generated using AF3, are shown in Figure 2. All five mutant proteins exhibited noticeable conformational alterations compared to the wild-type structure (Figure 2(a)). Specifically, the predicted RMSD values for each variant were as follows: c.161T > C (Figure 2(b)): 43.6 Å, indicating severe global structural disruption; c.335G > A (Figure 2(c)): 1.1 Å; c.1230_1238del (Figure 2(d)): 7.0 Å, suggesting substantial global deviation; c.482del (Figure 2(e)): 9.2 Å, also suggesting substantial global deviation; c.424_c.427delAAAGinsTTTCAGCAGCC (Figure 2(f)): 1.2 Å.

### Impact of novel *PAX2* variants on the proliferative capacity of HK-2 cells

**Figure 3.**
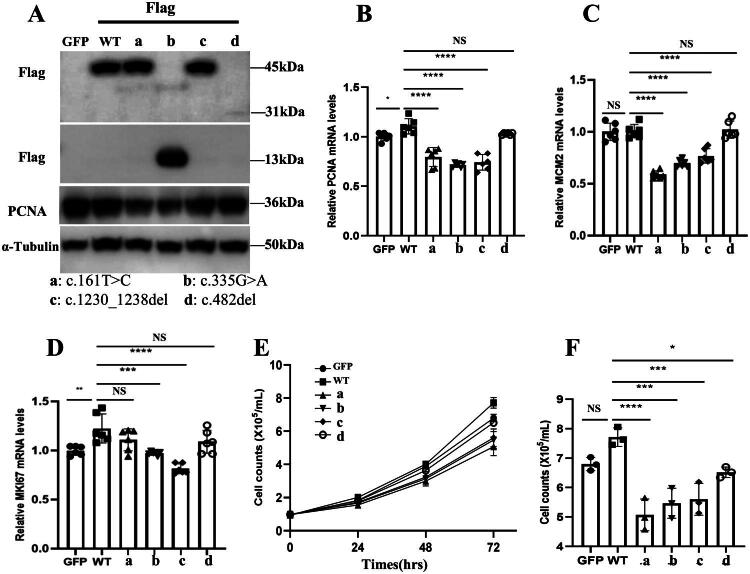
*PAX2* mutations inhibit cell proliferation in HK2 cells. (A) HK2 cells were transduced with various lentiviral vectors, including wild-type (WT) *PAX2*, mutational *PAX2* variants, and GFP. Western blot (WB) analysis was performed to detect the expression of Flag, PCNA, and α-Tubulin. (B) RT-qPCR analysis of mRNA levels of PCNA, (C) MCM-2, and (D) KI67 relative to β-Actin. (E) Cell growth curves illustrate the proliferation rates of HK2 cells with different *PAX2* mutations. (F) Cell count analysis of HK2 cells with different *PAX2* mutations after 72 hours of culture. Data are presented as mean ± standard deviation (SD). Statistical significance is indicated as *p < .05, **p < .01, ***p < .001, and ****p < .0001, determined by one-way ANOVA with Tukey’s multiple-comparisons test. NS: not significant.

According to the ACMG criteria, the c.424_c.427delAAAGinsTTTCAGCAGCC mutation identified in patient 10 is classified as pathogenic. The patient exhibited bilateral kidney dysplasia, which ultimately progressed to ESKD. Given the unequivocal pathogenicity of this variant, as supported by ACMG guidelines and the patient’s clinical phenotype, further functional validation was deemed unnecessary. Functional studies on other novel variants (c.161T > C, c.335G > A, c.1230_1238del, and c.482del) revealed that the *PAX2* protein molecular weight remained at 45 kDa for both c.161T > C and c.1230_1238del mutations, indicating that these mutations did not alter the overall molecular mass of the *PAX2* protein. However, the c.335G > A and c.482del mutations introduced premature stop codons in the CDS, leading to truncated *PAX2* proteins with molecular weights of approximately 13 kDa (363 bp) and 31 kDa (846 bp), respectively. Additionally, the c.482del mutation may compromise *PAX2* protein stability, triggering protein degradation and reducing its expression levels (Figure 3(A)). Further analysis revealed that both c.161T > C and c.335G > A mutations significantly decreased the expression of the proliferation marker PCNA protein (Figure 3(A)).

RT-qPCR results indicated that the c.161T > C mutation led to a decrease in the mRNA levels of PCNA and MCM2, but did not affect the expression of MKI67 mRNA (Figures 3(B–D)). Both the c.335G > A and c.1230_1238del mutations led to a significant reduction in the mRNA levels of cell proliferation markers PCNA, MCM2, and MKI67. Although the c.482del mutation did not significantly affect the mRNA levels of proliferation-related genes, cell proliferation assays revealed that all four new mutations reduced *PAX2*’s ability to promote cell proliferation (Figure 3(E,F)).

## Discussion

This study describes nine distinct *PAX2* variants in 10 pediatric patients, five of which are reported for the first time. Structural modeling using AF3 revealed varying degrees of conformational disruption associated with these novel mutations. We found that four novel variants may contribute to the development of kidney disease by impairing *PAX2*’s ability to regulate cell proliferation. Additionally, we report two novel phenotypes potentially associated with *PAX2* mutations, including unilateral hydrocele and liver dysfunction (hepatic fibrosis), which have not been previously described.

Among the 10 patients, four were found to have renal cysts, a prevalence consistent with reported rates in the literature [12,14]. Additionally, two patients in this study exhibited VUR. Previous studies have shown that *PAX2* mutations are one of the primary monogenic causes of VUR [18], and *PAX2* knockout mice also display prominent VUR features [19]. Deng et al. [20] suggested that kidney dysplasia and kidney cysts may be more common in China, whereas the incidence of VUR might be lower in Asians. However, a multicenter, systematic comparison remains lacking.

Studies have suggested that *PAX2* mutations associated with renal hypoplasia almost invariably progress to ESKD [21]. Okumura et al. [22] found that the proportion of *PAX2* mutation carriers requiring dialysis (54.5%) was significantly higher than that of non-mutated patients (13.3%). In our report, five patients progressed to stage 5 CKD between the ages of 3 and 11.1 years, with four of them undergoing allogeneic kidney transplantation, the average age at transplantation being only 10.4 ± 1.0 years. This indicates that children with *PAX2* mutations are at high risk of early progression to ESKD, and kidney transplantation serves as a viable and effective therapeutic strategy.

*PAX2* mutations are primarily linked to kidney and ocular abnormalities [3,23]. In our study, five patients had ocular abnormalities, while the majority exhibited isolated kidney structural or functional issues. Studies have shown that some individuals may present with isolated kidney abnormalities without overt ocular manifestations [24]. With the growing body of research on *PAX2*, additional associations have been discovered, including gallstones and testicular hypoplasia [24]. Furthermore, *PAX2* mutations may lead to abnormalities in auditory structures, which could be the underlying cause of hearing loss [25]. In our study, patient 10 exhibited hearing impairment. Additionally, we observed two rare extrarenal manifestations – right-sided hydrocele and liver dysfunction – which may expand the phenotypic spectrum of *PAX2*-related disorders. However, given the possibility that hepatic fibrosis and spermatic cord hydrocele are incidental, we cautiously propose these findings as potential novel phenotypes that warrant further investigation.

Studies have demonstrated that *PAX2* plays a crucial role in repairing acute kidney injury by promoting epithelial cell proliferation [26,27], and its re-expression is considered a marker of kidney injury repair [28]. During tumorigenesis, *PAX2* may also promote cancer cell proliferation and invasion by enhancing the formation of vascular-like structures [29]. Kidney dysplasia is primarily attributed to the loss of *PAX2*’s anti-apoptotic function, with *PAX2* haploinsufficiency increasing the susceptibility of ureteric bud cells to programmed cell death [30]. The increased apoptosis of ureteric bud cells reduces the number of ureteric bud branches, thereby affecting nephron formation [8,31]. Based on *PAX2*’s role in anti-apoptosis and promoting cell proliferation, we assessed the proliferation of HK2 cells transfected with newly identified mutations, including c.161T > C, c.335G > A, c.1230_1238del, and c.482del. Our results revealed a significant reduction in the proliferative capacity of these cells. AF3 structural modeling revealed distinct *PAX2* variant perturbations. Variants c.161T > C (RMSD = 43.6 Å) and c.482del (RMSD = 9.2 Å) caused severe structural disruption, including complete misfolding or truncation, likely eliminating regions essential for DNA binding and transcriptional activation. This correlates with their impaired ability to promote HK2 cell proliferation. The c.1230_1238del variant (RMSD = 7.0 Å) induced marked C-terminal changes within the transactivation domain, potentially disrupting interactions with co-factors required for proliferative signaling. Although variants c.335G > A and c.424_c.427del showed minimal global RMSD changes (1.1–1.2 Å), both caused localized distortions near or within the paired domain, affecting residues critical for DNA interaction or domain stability. These subtle but functionally important changes also resulted in reduced proliferative capacity in HK2 cells. Together, both extensive global misfolding and focal local disruptions in *PAX2* structure, as predicted by AF3 and quantified by RMSD, provide a mechanistic explanation for the impaired proliferation observed in cells expressing these mutants. We hypothesize that the mechanism underlying the development of kidney disease in patients with these *PAX2* mutations may involve a weakened ability of *PAX2* to regulate cell proliferation. This impairment may lead to a reduced number of ureteric bud branches, hinder normal kidney development, and ultimately result in the onset of kidney disease.

Our study has several limitations. First, this is a retrospective study, and only a subset of patients underwent eye examinations, hearing assessments, and retrograde pyelography, which limits our comprehensive evaluation of the extrarenal manifestations and the potential presence of reflux nephropathy. Additionally, we evaluated the impact of the mutations on tubular epithelial cell proliferation. While the results suggest a potential pathogenic role, the underlying mechanisms remain unclear and warrant further investigation, including validation in animal models and evaluation in complementary cell models.

## Conclusions

In conclusion, our study identified five novel *PAX2* mutations and functionally validated four of them. Additionally, we report liver dysfunction and spermatic cord hydrocele as potential novel phenotypes associated with *PAX2*-related diseases, thereby expanding the phenotypic spectrum of *PAX2* disorders. *PAX2* mutations significantly impact kidney function in children, emphasizing the importance of genetic testing and comprehensive renal function monitoring for early diagnosis and intervention, which are essential for improving prognosis.

## Supplementary Material

Answer to reviewers .docx

## Data Availability

The data presented in this study are available on request from the corresponding author due to privacy and ethical considerations.
