# Supplementary material for: Genotype–phenotype correlations and functional characterization of novel PAX2 variants in a 10-patient pediatric cohort
Source: Ren Fail. 2025 Sep 15;47(1):2552911. doi: 10.1080/0886022X.2025.2552911 (PMC12439803; doi:10.1080/0886022X.2025.2552911)
Supplement: Answer to reviewers .docx [file IRNF_A_2552911_SM2807.docx]

**Reviewer-1**

**Q1.** The study lacks detailed ophthalmological assessments of patients, merely describing visual field defects and amblyopia. I did not find any description of the specific ophthalmological examinations performed. The disease reported by the authors often presents with optic disc malformations and "morning glory" changes, which are crucial for diagnosis. Therefore, it is recommended that the authors perform more comprehensive fundus examinations to better characterize the ocular manifestations of this disease.

R1. We sincerely thank the reviewer for the valuable comments. Patients with *PAX2* mutations typically undergo fundoscopic examination and optical coherence tomography (OCT) to evaluate optic disc morphology (e.g., hypoplasia, excavation) and retinal vascular abnormalities (e.g., tortuosity, neovascularization). The “morning glory disc anomaly” is a distinctive but not universally present ocular malformation, with its prevalence varying across different cohorts. Notably, we did not observe typical “morning glory” signs in any patients within our cohort. In our retrospective cohort, relatively complete ophthalmological assessments—including fundoscopy and OCT—were available for six patients. Abnormal findings included amblyopia, visual field defects, indistinct optic disc margins, enlarged optic cup, and enlarged physiological blind spots. We have added this information to the revised Results section. As this was a retrospective study, we were unable to obtain complete fundoscopic and OCT data for all 10 patients. This limitation has indeed restricted our ability to fully characterize the ocular phenotype spectrum in this cohort. We have addressed this issue as an inherent challenge of retrospective studies in the revised Discussion section.

**Q2.** The authors evaluated whether the mutations in this patient group were novel using the Human Gene Mutation Database (HGMD) and ClinVar. However, there is currently a specialized *PAX2* mutation database that is continuously updated (http://www.lovd.nl/*PAX2*). It is recommended that the authors refer to this database to assess the mutation types and clinical manifestations in this patient group.

**R2**. Thank you for pointing this out. We have now included the Leiden Open Variation Database (LOVD) for *PAX2* ([http://www.lovd.nl/*PAX2*](http://www.lovd.nl/PAX2)) in our variant assessment. A comparison of our detected variants with those listed in LOVD has been added in the revised “Results” section, and a corresponding description is included in the “Methods.”

**Q3**. Regarding statistical methods, the authors used mean ± standard deviation for all data. The normality of data distribution and homogeneity of variance should be determined first before selecting appropriate statistical description methods.

**R3**. Thank you for your suggestion. We have now performed Shapiro–Wilk tests to assess the normality of data distribution and Levene’s tests for homogeneity of variance. Data that were not normally distributed are now reported using median and interquartile range. The revised statistical analysis method has been detailed in the “Statistical Analysis” section.

**Q4**. The experimental validation section is overly simplistic, and the obtained results do not provide meaningful references for future diagnosis and treatment of this disease. The research significance is limited.

**R4.** We sincerely thank the reviewer for their valuable comments. In this study, we constructed lentiviral vectors carrying different *PAX2* mutants and transfected them into HK-2 renal tubular epithelial cells. Western blot analysis showed that the c.161T>C and c.1230_1238del variants did not alter the molecular weight of the *PAX2* protein (~45 kDa), whereas the c.335G>A and c.482del variants introduced premature stop codons, resulting in truncated proteins of approximately 13 kDa and 31 kDa, respectively, suggesting impaired protein stability. Furthermore, we utilized AlphaFold 3（AF3）to predict the three-dimensional conformations of the five *PAX2* variant proteins. Quantitative comparison using Root Mean Square Deviation (RMSD)^1,2^ revealed varying degrees of conformational disruption in all mutant proteins, with marked structural deviations from the wild-type protein (Figure 3). At the cellular functional level, proliferation assays demonstrated that all novel variants significantly impaired the ability of *PAX2* to promote cell proliferation. In conjunction with the structural modeling results, we hypothesize that the conformational perturbations caused by *PAX2* mutations may directly lead to loss of function, thereby impairing the proliferative capacity of renal epithelial cells. We further propose that this reduced proliferative ability may interfere with ureteric bud branching during early embryonic development, disrupting normal kidney morphogenesis and offering a potential explanation for the renal dysplasia observed in mutation carriers.

We acknowledge that our current validation experiments remain preliminary and have not yet explored the specific regulatory effects of *PAX2* variants on downstream target genes or signaling pathways. However, the integration of protein structural modeling and functional cell-based assays provides a reasonable and initial line of evidence supporting the pathogenic potential of these novel variants. In future studies, we plan to employ animal models and renal progenitor cell lines to systematically assess the developmental impact of distinct *PAX2* mutations and to further elucidate their underlying molecular mechanisms. We believe that these preliminary findings establish a conceptual basis for future mechanistic studies and contribute to advancing functional diagnostics of *PAX2*-related disorders.

**Q5**. The research objectives of this study are unclear, and the discussion section fails to adequately discuss the findings of this study. The overall writing quality needs improvement.

**R5**. We sincerely appreciate the reviewer’s insightful comments. In response, we have thoroughly revised the *Introduction* and *Discussion* to better clarify the study objectives and enhance the interpretation of our results. Furthermore, we have carefully edited the entire manuscript, including the abstract, to improve its overall readability and language quality.

**Q6**. The figures in the manuscript lack figure legends.

**R6**.We apologize for the oversight. Figure legends have now been added to all figures to ensure clarity and completeness.

**Reviewer-2**

Comments

| **Manuscript section** | **Description** |
| --- | --- |
| Title | It cleary conveys the topic of the study and indicates type of study. |
| Abstract | • The background and objectives of the study were well presented in the abstract. However, a brief descriptions of data collection tools and data analysis need to be added to the Methods Section of the Abstract.  • The key findings of, as well as the conclusion(s) drawn from, the study were mentioned in the Abstract.  • Moreover, keywords were provided, but need to be arranged alphabetically. |
| Introduction | • The authors adequately explained the genetics, the physiology as well as the clinical impact of *PAX2* genes.  • The pathological manifestations of *PAX2* gene disorders were clearly explained by the authors.  • The rationale for conducting the study as well as the aim of study were clearly stated by the authotrs. |
| Methods | • The study design and setting were clearly stated.  • The study variables as well as data collection methods  were clearly stated and adequately explained.  • Data analysis was clearly described.  • Ethical Approval was stated. |
| Results | • The authors presented the findings of study with adequate clarification. |
| Discussion | • The authors discussed the findings of the study in light of, and integrated them with, relevant prior work.  • The implications of the study findings for paediatrics speciality, their contribution to the field as well as trasferability of results were adequately addressed in the Discussion.  • The author(s) provided an interpretation of study data with reference to study objectives. |
| Limitations of study | These were obviously stated and they were relevant. |
| Conclusions | These were clearly stated in line with the aims of study as well as data presented in current study. |
| References | These are up-to-date, relevant and written in an appropriate style. |
| Conflict of interest | Clearly stated. |
| Funding of study | Obviously declared. |
| Overall comments | The manuscript is interesting as it has explored an important topic in paediatrics. In addition, it is well-written and adequately-referenced. |

**R.** We thank the reviewer for their recognition and constructive suggestions. In response, we have added a brief description of the data collection tools and data analysis methods in the *Methods* section of the abstract. Additionally, the keywords have been reordered alphabetically.

**Reviewer-3**

**Q1**. While hepatic dysfunction and spermatic cord hydrocele are reported as novel extrarenal manifestations of *PAX2*-related disease, the authors should address whether these findings represent true phenotypic expansions or are coincidental comorbidities unrelated to the genetic variant. Please add it in the discussion. A more detailed discussion would strengthen the interpretation of these findings.

**R1**. Thank you for this important comment. We have now discussed the possibility that hepatic fibrosis and spermatic cord hydrocele may be incidental findings unrelated to *PAX2* variants. However, given the absence of other identifiable causes in these patients, we cautiously propose them as potential novel phenotypes requiring further validation. This has been added to the “Discussion” section.

**Q2**. In the discussion section, the authors state that “we assessed the effect of mutations on the proliferative function of tubular epithelial cells; however, the underlying mechanism warrants further investigation.” What is the potential mechanistic links between cellular findings and clinical phenotypes? Are there any testable hypotheses?

**R2**. Thank you for raising this point. The *PAX2* gene is widely expressed in key structures of metanephric development, including the metanephric mesenchyme (MM), ureteric bud (UB), and renal vesicles. It promotes UB-derived signaling molecules, inducing mesenchymal cell condensation and differentiation into nephrons. Studies demonstrate that *Pax2* knockout in mice significantly reduces UB branching and nephron formation while markedly increasing apoptosis during renal development**^3^** ^4^. As a transcription factor, *PAX2* primarily orchestrates kidney development by regulating downstream target gene expression. For instance, the nephrogenic molecules WT1 and GDNF, which are indispensable for metanephric induction and growth within the MM, are transcriptionally regulated by *PAX2*^5^. Jiang et al. ^6^ proposed that *PAX2* modulates renal development by specifically binding to the TBX1 promoter to regulate its expression. Furthermore, Yamamura et al. ^7^ identified PBX1, POSTN, and ITGA9 as crucial downstream effectors of *PAX2* during human and murine kidney development. Notably, *Pax2* deficiency suppresses the expression of nephron-associated genes (e.g., SIX2, CRYM, EYA1) while activating genes associated with interstitial cells (e.g., MEIS1, ANXA2, FOXD1) ^8^. Collectively, these findings suggest that *PAX2* mutations may drive diverse disease phenotypes by dysregulating downstream effectors, disrupting cellular proliferation processes, and inducing structural maldevelopment**.** Our preliminary evidence indicates that *PAX2* mutation significantly attenuates the proliferative capacity of human renal proximal tubular epithelial (HK-2) cells. To elucidate the underlying mechanisms, we will employ nephron progenitor cell (NPC) models in subsequent investigations.

**Q3**. The manuscript is generally well written, though there are a few minor lapses in adherence to reporting standards. For example, the sentence “This study included a total of 10 patients, with a male-to-female ratio of 6:4” should be revised to report the exact numbers, as recommended by the ICMJE guidelines.

**R3**. Thank you for noting this. We have revised the sentence to: “This study included a total of 10 patients, including 6 males and 4 females,” in accordance with ICMJE guidelines.

**Reviewer-4**

**Q1**.The presentation of institutional affiliations differs between the title page and the Materials and Methods section.

**R1**. Thank you. We have carefully revised and unified the institutional affiliations in both the title page and the Methods section to ensure consistency.

**Q2**.Multiple instances of non-standard gene nomenclature formatting are present in the manuscript. *PAX2* should be consistently typeset in italics throughout the text.

**R2**. We apologize for this formatting error. All gene symbols including *PAX2* have now been consistently italicized throughout the manuscript.

**Q3**.A discrepancy exists between the statistical methodology description ('Data are expressed as mean ± standard error of the mean' in Methods) and the graphical presentation ('mean ± SD' in Figure 2F legend).

**R3**. Thank you. We have corrected the inconsistency and now report all data as mean ± standard deviation (SD) consistently across text and figures. This is clarified in both the Methods section and figure legends.

**Q4**.The limited clinical cohort size in this study precludes definitive conclusions regarding a causal relationship between *PAX2* variants and the reported novel phenotypes of hepatic fibrosis and spermatic cord hydrocele, as current evidence remains insufficient to establish mechanistic links.

**R4**.Thank you for the reviewer’s insightful comment. In our study, although we excluded other known causes of hepatic fibrosis and spermatic cord hydrocele, the limited sample size prevents us from drawing a definitive causal link between *PAX2* variants and these potential novel phenotypes. We have acknowledged this limitation and added it to the *Discussion* section.

**Q5**.Beyond the HK-2 cell line used in this study, were more developmentally relevant renal cell models—such as human embryonic kidney cells or nephron progenitor cells—considered for functional validation of *PAX2* variants?

**R5:** We appreciate the reviewer’s valuable suggestions. In the present study, we selected the HK-2 cell line based on two main considerations:

**1.Model Reliability:** HK-2 cells exhibit immortalization and retain key functional characteristics of human proximal tubular epithelial cells, including anchorage dependence, gluconeogenesis capability, and sodium-dependent sugar transport^9,10^. Morphological and functional comparisons have demonstrated that HK-2 cells closely resemble primary human proximal tubular epithelial cells^11^, which has led to their widespread use as a classic in vitro model for investigating renal pathophysiology.

**2.Experimental Suitability:** Our study aimed to preliminarily assess the effects of *PAX2* mutations on cell proliferation. HK-2 cells possess stable proliferation characteristics and high transfection efficiency, thereby providing an ideal experimental platform for quantitative analyses of the impact of *PAX2* variants on cellular proliferative capacity.

Nevertheless, we fully acknowledge that relying solely on an adult tubular epithelial cell model has inherent limitations in recapitulating the developmental biology of *PAX2* in kidney morphogenesis. To address this limitation, we are actively pursuing further studies that will incorporate additional cell models—such as nephron progenitor cells and primary tubular epithelial cells—to perform more developmentally relevant functional assessments and mechanistic investigations. We believe that these efforts will enhance our understanding of the role of *PAX2* mutations in renal developmental abnormalities and disease pathogenesis.

**Reference**

1. Bagaria A, Jaravine V, Huang YJ, Montelione GT, Güntert P. Protein structure validation by generalized linear model root-mean-square deviation prediction. *Protein science : a publication of the Protein Society.* 2012;21(2):229-238.

2. Rettie SA, Campbell KV, Bera AK, et al. Cyclic peptide structure prediction and design using AlphaFold. *bioRxiv : the preprint server for biology.* 2023.

3. Dziarmaga A, Eccles M, Goodyer P. Suppression of ureteric bud apoptosis rescues nephron endowment and adult renal function in Pax2 mutant mice. *Journal of the American Society of Nephrology : JASN.* 2006;17(6):1568-1575.

4. Longaretti L, Trionfini P, Brizi V, et al. Unravelling the Role of *PAX2* Mutation in Human Focal Segmental Glomerulosclerosis. *Biomedicines.* 2021;9(12).

5. Grote D, Souabni A, Busslinger M, Bouchard M. Pax 2/8-regulated Gata 3 expression is necessary for morphogenesis and guidance of the nephric duct in the developing kidney. *Development (Cambridge, England).* 2006;133(1):53-61.

6. Jiang H, Li L, Yang H, Bai Y, Jiang H, Li Y. Pax2 may play a role in kidney development by regulating the expression of TBX1. *Molecular biology reports.* 2014;41(11):7491-7498.

7. Yamamura Y, Furuichi K, Murakawa Y, et al. Identification of candidate *PAX2*-regulated genes implicated in human kidney development. *Scientific reports.* 2021;11(1):9123.

8. Naiman N, Fujioka K, Fujino M, et al. Repression of Interstitial Identity in Nephron Progenitor Cells by Pax2 Establishes the Nephron-Interstitium Boundary during Kidney Development. *Developmental cell.* 2017;41(4):349-365.e343.

9. Mossoba ME, Sprando RL. In Vitro to In Vivo Concordance of Toxicity Using the Human Proximal Tubule Cell Line HK-2. *International journal of toxicology.* 2020;39(5):452-464.

10. Huang F, Wang Q, Guo F, et al. FoxO1-mediated inhibition of STAT1 alleviates tubulointerstitial fibrosis and tubule apoptosis in diabetic kidney disease. *EBioMedicine.* 2019;48:491-504.

11. Prange JA, Bieri M, Segerer S, et al. Human proximal tubule cells form functional microtissues. *Pflugers Archiv : European journal of physiology.* 2016;468(4):739-750.
